# Validation of the International Weed Genomics Consortium genome annotation pipeline through reannotation of the model species *Arabidopsis thaliana*

**DOI:** 10.1002/tpg2.70270

**Published:** 2026-06-30

**Authors:** Luan Cutti, Daniel Fernando da Silva Filho, Geisson Edwin Guadir Lara, Jessica Matheson, Nicholas A. Johnson, Jacob Montgomery, Nathan Hall, Brent Murphy, Todd A. Gaines, Eric L. Patterson

**Affiliations:** ^1^ Department of Plant, Soil, and Microbial Sciences Michigan State University East Lansing Michigan USA; ^2^ Department of Biological Sciences São Paulo State University Bauru São Paulo Brazil; ^3^ Department of Biology University of Massachusetts Amherst Amherst Massachusetts USA; ^4^ BASF SE Agricultural Research Station Limburgerhof Germany; ^5^ Department of Agricultural Biology Colorado State University Fort Collins Colorado USA

## Abstract

The International Weed Genomics Consortium (IWGC) has sequenced and annotated the genomes of over 30 weed species, generating genomic resources to understand their biology, evolution, and adaptation. The objective of this study was to evaluate the semi‐automated, isoform sequencing (Iso‐seq)‐based, IWGC genome annotation pipeline by reannotating the genome of the model species *Arabidopsis thaliana* with various amounts and types of extrinsic data and to measure the impact that varying inputs had on the annotation completeness and quality. Annotations were run comparing the effects of (1) the quantity and source of Iso‐seq reads, (2) annotated proteins from botanically closely related or distantly related species, and (3) the number of proteins provided to the annotation program “*MAKER‐P*.” Reannotations were compared to each other and to the published annotation of the *A. thaliana* genome. The IWGC annotation pipeline annotated almost all the genes without manual curation when informed with an Iso‐seq dataset and proteins of related species. In general, the pipeline produced more accurate, annotated genes with more input proteins, especially from closely related species, in the gene model prediction step. Furthermore, the combination of proteins from several closely related species increased the number of annotated genes. The number or source of Iso‐seq reads did not have a significant effect if many proteins from closely related species were utilized. The annotation pipeline annotated nearly 90% of genes from additional crop species genomes. The IWGC genome annotation pipeline is robust in reannotating the *A. thaliana* genome and therefore is most likely performing well in the several non‐model weed species it has been used on so far.

## Plain Language Summary

The International Weed Genomics Consortium (IWGC) is generating genome resources for weeds, but accurate gene annotation is essential because most weeds are not model plants. To test their annotation pipeline, we reannotated the well‐studied genome of *Arabidopsis thaliana*. We evaluated how different supporting data, such as long‐read transcript (isoform sequencing [Iso‐seq]) data and protein sequences from related and distant species, affected gene prediction. The pipeline successfully identified nearly all genes without manual correction when supported by Iso‐seq data and proteins from related species. Adding more protein sequences, especially from closely related species, improved accuracy and gene recovery. In contrast, increasing the amount or changing the source of Iso‐seq data had little additional effect when sufficient related protein data were available. Overall, the IWGC pipeline produces reliable, high‐quality genome annotations.

**Abbreviations**
BUSCO: Benchmarking Universal Single‐Copy Orthologs
CDS: coding sequence
Iso‐seq: isoform sequencing
IWGC: International Weed Genomics Consortium
NCBI: National Center for Biotechnology Information
PacBio: Pacific Biosciences
Pfam: protein families database
PSAURON: protein structure audit oriented evaluation

## 1 INTRODUCTION

Out of at least 4604 plant genomes from 1482 species sequenced to date (Bernal‐Gallardo & de Folter, 2024), only a few are high‐impact agricultural weed species. The first relevant weed genome published was *Echinochloa crus‐galli* in 2017 (Guo et al., 2017), followed by several other species in recent years, with about 32 relevant weed species currently having sequenced reference genomes available (Chen et al., 2024). The International Weed Genomics Consortium (IWGC) is a public and private collaboration to sequence, assemble, and annotate the reference genomes from a majority of the most important international weeds, including diploid and polyploid weed species; examples include *Amaranthus palmeri* (diploid; 0.44 Gb), *Lolium rigidum* (diploid; 2.41 Gb), *Chenopodium album* (allohexaploid; 1.59 Gb), and *Avena fatua* (allohexaploid; 11.2 Gb) (Montgomery et al., 2024). The IWGC has sequenced and annotated de novo genomes from over 30 species and reannotated several other publicly available genomes sequenced by other research groups (International Weed Genomics Consortium, 2025). Furthermore, for several of the most important species, multiple haplotypes and, in some cases, distinct individuals were analyzed, allowing us to begin exploring pan genomic variation.

One of the most pressing uses of these weed genomes is to study herbicide resistance mechanisms and their evolution. Evolved herbicide resistance mechanisms in weeds range from genetically simple, such as single nucleotide polymorphisms in the genes coding herbicide target proteins, to more complex, such as those involving genomic structural variation or enzymes exhibiting substrate promiscuity that can detoxify herbicides. The most common gene families involved in herbicide metabolism are the *cytochrome P450 monooxygenase*, *glutathione‐S‐transferase*, and *ATP‐binding cassette* families (Gaines et al., 2020), which each have hundreds of gene members with high identity (especially in allopolyploids compared to diploids), such as in *E. crus‐galli* (Wu et al., 2022). These large and highly relevant gene families make accurate annotations crucial for genome‐wide studies such as RNA sequencing or genome‐wide association study, where researchers are looking for herbicide resistance candidate genes. Accurate gene annotations will also allow researchers to find novel enzymes for herbicide detoxification, transport, or new herbicide targets.

Genome structure can also be a critical component of understanding traits related to species adaptation, biology, and herbicide resistance. For example, recently, a transposable element insertion in *indole‐3‐acetic acid 16* in *Bassia scoparia* changes normal gene splicing and the altered coding sequence (CDS) confers resistance to dicamba (an auxinic herbicide, Group 4) (Montgomery et al., 2025). In other cases, the co‐duplication of chromosome segments carrying herbicide target genes into extra‐chromosomal circular DNA confers glyphosate (Molin et al., 2020) and glufosinate‐ammonium (Carvalho‐Moore et al., 2025) resistance in *A. palmeri*, while a tandem duplication of a herbicide target gene in the subtelomeric region of *Eleusine indica* confers herbicide resistance (C. Zhang et al., 2023). Beyond herbicide resistance studies, a better understanding of weed biology also requires adequate genomic information. *Amaranthus palmeri* and *Amaranthus tuberculatus*, two of the major problematic weed species, are dioecious, and de novo genome assembly has been utilized to identify the sex‐determining region and specific genes involved in sex determination (Raiyemo, Cutti, et al., 2025; Raiyemo, Montgomery, et al., 2025). Most of these unique results utilized IWGC‐produced genomes in their studies, highlighting the need and importance of high‐quality reference genomes to advance weed science and generate new tools for weed control, such as RNAi and gene drive (Neve, 2018; Panozzo et al., 2025; Zabala‐Pardo et al., 2022).

The IWGC uses the most cutting‐edge sequencing available to assemble these weedy genomes, using the combined approaches of Pacific Biosciences (PacBio) HiFi reads, ultra‐long Oxford Nanopore data, Hi‐C (high‑throughput chromosome conformation capture) contact mapping, and optical mapping. This has resulted in low‐gap chromosome‐level assemblies, many of which are haplophased (Gomes et al., 2025; J. Lemas, Ņečajeva, et al., 2025; J. M. Lemas, Patterson, et al., 2025; Raiyemo, Cutti, et al., 2025; Raiyemo, Montgomery, et al., 2025). For gene model prediction, the IWGC has primarily utilized isoform sequencing (Iso‐seq) reads (PacBio HiFi of full‐length RNA molecules), which are expected to make the gene model and splice form prediction more accurate than when utilizing short reads. In the IWGC annotation pipeline, evidence such as Iso‐seq reads and protein sequences from closely related species, called extrinsic evidence, are combined with computational predictions by statistical models, called intrinsic evidence, to support final gene model prediction. The utilization of only extrinsic evidence may not cover the whole genome, while only intrinsic evidence is error‐prone, which makes the combination of extrinsic and intrinsic evidence a flexible approach for accurate genome annotation (Brůna et al., 2020; Gabriel et al., 2024).

Genome annotation is an extensive and iterative process, with its complexity escalating proportionally to the genome's size and complexity. This process relies on the sequential integration of software, algorithms, and methodologies, alongside access to accurate and up‐to‐date sequence databases. Implementing a pipeline that integrates various programs minimizes the need for time‐consuming and labor‐intensive manual curation. The objective of this study was to develop a semi‐automated genome annotation pipeline and validate it by reannotating the genome of the model species *Arabidopsis thaliana* with various amounts and types of extrinsic data to measure the types of errors the pipeline generates, where the best return on investment is to be found for data generation, and ultimately understand the bias that imperfect genome annotation has on downstream analysis that relies on these reference genomes. With this paper, we also make the IWGC annotation pipeline easily accessible through a Singularity distribution environment.

## 2 MATERIALS AND METHODS

### 2.1 The IWGC annotation pipeline

**FIGURE 1:**
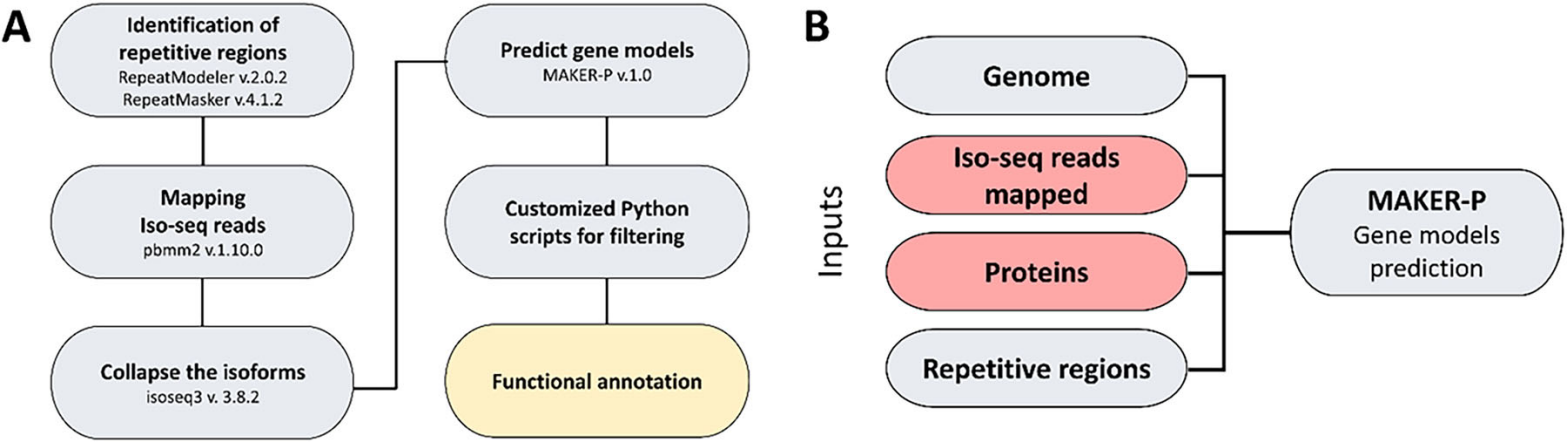
(A) International Weed Genomics Consortium genome annotation pipeline. (B) Files input in *MAKER‐P* step, highlighting in red the two variable inputs that were utilized to build the different annotation scenarios

The IWGC genome annotation pipeline integrates pre‐existing publicly available software tools. First, genome assemblies have the genome‐wide de novo transposable element families identified using *RepeatModeler v.2.0.2* (Flynn et al., 2020), annotated using *RepeatMasker v.4.1.2* (Flynn et al., 2020; Smit et al., 2025), and soft masked using *BEDtools v.2.30.0* (Quinlan & Hall, 2010). The IWGC generates Iso‐seq reads, full‐length transcript isoforms with no assembly required, from leaves, flowers, roots, and stem to support the annotation. The Iso‐seq reads are aligned to the softmasked genome utilizing the *pbmm2 v. 1.10.0* part of the official Isoseq3 pipeline, and the transcript isoforms are collapsed into a single representative using *isoseq3 v.3.8.2* (PacBio & Bioconda, 2025). Gene model prediction is performed by *MAKER‐P v.1.0* (Campbell, Law, et al., 2014), utilizing proteins from closely related species to support the gene model prediction. Small, predicted proteins (<35 amino acids) are filtered out following internal customized Python scripts (Figure 1A). The functional annotation of the gene models utilizes *MultiLoc2 v.1.0* (Blum et al., 2009), *SignalP* (Teufel et al., 2022), and *TargetP* (Armenteros et al., 2019) to predict the intracellular location of the protein; *HMMER v.3.3* (Eddy, 2008) and *Pfam* (protein families database) (Mistry et al., 2021) to search for protein domain homology; *iprscan5 v.5.47‐82.0* (Jones et al., 2014) to search in the InterPro collection of protein signature databases; *MMSeqs2 v.4.1* (Steinegger & Söding, 2017) to search in UniRef50 from UniProt and a custom National Center for Biotechnology Information (NCBI) protein database; and the best hit of UniRef50 was converted into the Kyoto Encyclopedia of Genes and Genomes Ortholog ID (Kanehisa et al., 2017).

### 2.2 IWGC pipeline automation

To automate the IWGC genome annotation pipeline, we utilized containerization, specifically Docker and Singularity, to encapsulate the necessary bioinformatics tools and their dependencies. The entire workflow was managed using Snakemake, a workflow management system that provides a scalable framework for bioinformatics pipelines (Mölder et al., 2021). Snakemake simplifies pipeline automation and processing of bioinformatic data and enables the creation of isolated environments for each bioinformatics tool using Conda, Docker (Merkel, 2014), or Singularity (Kurtzer et al., 2017), automating the installation and the use of the entire pipeline. The annotation pipeline was divided into two main components, “structural” and “functional” annotation. Each component utilized specific Docker images corresponding to the required tools (Supporting Information). Docker images were used to generate Singularity containers that were orchestrated to run sequentially, with outputs from one tool seamlessly fed into the next, managing data exchange and maintaining consistent computational environments. This coordination simplified user interaction and allowed for customizable configurations, such as resources and databases, between runs. For each part, we employed a dedicated Snakefile, a configuration file, and a structured folder containing scripts, images, and input files specific to attend to the requirements of that phase. In Snakemake, each analytical step was defined as a discrete rule specifying the required Singularity container, inputs, outputs, and computational commands. Containerization was incorporated directly within each rule through the Singularity directive, thereby aligning each processing step with the appropriate software dependencies. Resource management, including job scheduling, parallelization, and computational resource allocation, was seamlessly handled by Snakemake, optimizing the pipeline's flexibility across available computational resources. Minimal user configuration was required, limited to defining input files, resource parameters, and essential databases, thus streamlining pipeline deployment and facilitating its adaptability to various datasets and computational environments. Snakemake autonomously orchestrates the execution order and dependency resolution. The whole pipeline and scripts are available on the GitHub page (see Data Availability Statement section).

The output from structural annotation is a filtered GFF3 (General Feature Format version 3) file that provides a detailed and hierarchical representation of the genomic features identified in the species under study. This file includes essential annotations such as genes, messenger RNAs, exons, CDSs, untranslated regions, and transfer RNAs, offering a refined and organized annotation optimized for further analysis and interpretation in a standard format. The output from the functional annotation step is encapsulated in a TSV (tab‐separated value) file, which presents a comprehensive summary of gene annotations from multiple bioinformatics tools and databases, allowing a detailed view across various functional categories, including subcellular localization predictions, gene ontology terms, protein family classifications, and additional functional descriptors.

### 2.3 Validation of the IWGC annotation pipeline using *A. thaliana*

A published genome of model species *A. thaliana* Col‐0 (Hou et al., 2022) was utilized to validate the IWGC annotation pipeline as it is probably the most studied and complete of any plant genome and the closest representation of a completely annotated genome. Furthermore, there is a plethora of publicly available datasets from different tissue types, environmental conditions, and sister species. Validation consisted of comparing the number of genes annotated in the published *A. thaliana* genome (Hou et al., 2022) with different input scenarios in our annotation pipeline, such as Iso‐seq reads from different tissues and stresses, different number of Iso‐seq reads, proteins from different species, different number of proteins, and their combinations. Different Iso‐seq reads scenarios were utilized in the mapping step (*pbmm2*) and were obtained from different *A. thaliana* tissues (flowers, leaves, roots, and siliques) and also from different abiotic (cold, heat, and flooding) and biotic (*Botrytis cinerea*, *Pseudomonas syringae*, and *Hyaloperonospora arabidopsidis*) stresses. The Iso‐seq reads from the individual tissues and stresses were combined to create four samples: “Bulk_1” (leaves + flowers + roots); “Bulk_2” (leaves + flowers + roots + siliques + seedlings); “Bulk_3” (leaves + flowers + roots + siliques + seedlings + cold + heat + flooding); and “Bulk_4” (leaves + flowers + roots + siliques + seedlings + cold + heat + flooding *+ B. cinerea* + *P. syringae* + *H. arabidopsidis*) (Table 1 and Table S1). These different Iso‐seq reads samples utilized in the mapping step were combined with different sources and amounts of proteins utilized as input in the gene model prediction step (*MAKER‐P*). The annotated proteins from *A. thaliana*, *Brassica napus*, the combination of *Arabidopsis arenosa* + *Arabidopsis suecica* + *B. napus* + *Camelina sativa*, or no proteins were utilized. The number of genes annotated running the IWGC pipeline was compared to the total of 27,561 genes from the *A. thaliana* genome paper (Hou et al., 2022), which was considered 100%.

**TABLE 1:** *Arabidopsis thaliana* isoform sequencing (Iso‐seq) read sources (tissues, and abiotic and biotic stresses) utilized in annotation pipeline validation.

Sample	Number of Iso‐seq reads	Sample description
Leaves	1,381,147	Leaves
Flowers	1,965,639	Flowers
Roots	7,789,289	Roots
Siliques	12,071,419	Siliques
Bulk_1	11,136,075	(Leaves + flowers + roots)
Bulk_2	19,735,858	(Leaves + flowers + roots + siliques + seedlings)
Bulk_3	45,349,042	(Leaves + flowers + roots + siliques + seedlings) + (cold + heat + flooding)^a^
Bulk_4	61,392,270	(Leaves + flowers + roots + siliques + seedlings) + (cold + heat + flooding) + (infected by *Botrytis cinerea* + *Pseudomonas syringae* + *Hyaloperonospora arabidopsidi*s)^a^

^a^ The complete biotic and abiotic conditions are described in R. Zhang et al. (2022).

“Bulk_2” with a total of 19,735,858 Iso‐seq reads was used to evaluate the effect of decreasing the number of Iso‐seq reads on the number of genes annotated. The annotation pipeline was run with different, random, “Bulk_2” subsets (100%, 80%, 60%, 40%, 20%, 10%, 5%, 1%, and 0.5%) in the mapping step (Table 2). The annotation utilizing each Iso‐seq subset was also run utilizing different protein inputs from *A. thaliana*, *B. napus*, or the combination of *A. arenosa* + *A. suecica* + *B. napus* + *C. sativa*. In addition, to evaluate the influence of the number of proteins utilized as input in the gene model prediction step, proteins from *A. arenosa* + *A. suecica* + *B. napus* + *C. sativa* were combined in a file with 314,397 proteins and were also subset (100%, 80%, 60%, 40%, 20%, 10%, 5%, 1%, and 0.5%), and the annotation pipeline was run utilizing “Bulk_2” as the source of Iso‐seq reads in the mapping step. These data were fitted to a three‐parameter sigmoidal curve using the *drc* package in *R* (Ritz et al., 2015).

**TABLE 2:** Number of isoform sequencing (Iso‐seq) reads and proteins of each subset.

Subset	Number of Iso‐seq reads^a^	Number of proteins^b^
Subset 100%	19,735,858	314,397
Subset 80%	15,788,610	251,356
Subset 60%	11,839,901	188,657
Subset 40%	7,894,582	125,759
Subset 20%	3,949,857	63,126
Subset 10%	1,974,803	31,599
Subset 5%	987,070	15,742
Subset 1%	197,443	3113
Subset 0.5%	98,528	1548

^a^ Iso‐seq reads subset 100% refers to the Bulk_2 (leaves + flowers + roots + siliques + seedlings).

^b^ Proteins subset 100% refers to the combination of proteins from *A. arenosa + A. suecica + B. napus + C. sativa*.

To evaluate whether Iso‐seq reads or protein inputs are more critical for the gene model prediction, their subsets of 10% and 80%, described before, were combined (10% proteins + 10% Iso‐seq reads; 80% proteins + 80% Iso‐seq reads; 10% proteins + 80% Iso‐seq reads; and 80% proteins + 10% Iso‐seq reads) to run the pipeline. The 10% and 80% subsets were chosen to represent low‑ and high‑evidence annotation conditions. To evaluate the influence of taxonomic proximity of the protein on gene model prediction data, proteins from different species—*A. thaliana*, *A. suecica*, *A. arenosa*, *C. sativa*, *Capsella rubella*, *B. napus*, *Glycine max*, *Cucumis melo*, *Gossypium laxum*, *Amborella trichopoda*, *Oryza sativa*, *Ceratodon purpureus*, and *Chlamydomonas schloesseri*—representing a range from botanically close to distant (respectively) to *A. thaliana* (Table 3)—were utilized in the gene model prediction step (*MAKER‐P*). A phylogenetic tree was generated from protein sequences of all species, and the resulting topology, derived using OrthoFinder (Emms & Kelly, 2019) and visualized in iTOL (https://itol.embl.de/), was used to rank species by their botanical proximity to *A. thaliana* (Figure S1). The number of genes annotated was counted at the end of the pipeline. In addition, the combinations of proteins from more closely related species *C. sativa + C. rubella + B. napus* (261,957 proteins total), and from more distantly related species *G. laxum + C. melo + G. max* (138,577 proteins total), were utilized to run the pipeline for comparison. These taxonomic proximity annotations were run utilizing Bulk_2 (from flowers + leaves + roots + siliques + seedlings) as the source of Iso‐seq reads in the mapping step.

**TABLE 3:** Number of proteins and botanical information of different species utilized as protein sources in the annotation.

Species	Family	Tribe	Genus	Number of proteins
*Arabidopsis thaliana*	Brassicaceae	Camelineae	*Arabidopsis*	48,359
*Arabidopsis arenosa*	Brassicaceae	Camelineae	*Arabidopsis*	23,097
*Arabidopsis suecica*	Brassicaceae	Camelineae	*Arabidopsis*	63,469
*Camelina sativa*	Brassicaceae	Camelineae	*Camelina*	107,481
*Capsella rubella*	Brassicaceae	Camelineae	*Capsella*	34,126
*Brassica napus*	Brassicaceae	Brassiceae		120,350
*Glycine max*	Fabaceae			74,248
*Cucumis melo*	Cucurbitaceae			35,817
*Gossypium laxum*	Malvaceae			28,512
*Amborella trichopoda*	Amborellaceae			31,494
*Oryza sativa*	Poaceae			42,580
*Ceratodon purpureus*	Ditrichaceae			40,806
*Chlamydomonas schloesseri*	Chlamydomonadaceae			16,268

To evaluate if transcripts from different species could replace the *A. thaliana* Iso‐seq reads, the annotation was run utilizing proteins and transcripts from *B. napus* (152,582 transcripts), *C. rubella* (37,318 transcripts), *C. sativa* (123,030 transcripts), and their combination (*B. napus* + *C. rubella* + *C. sativa* = 312,930 transcripts) to annotate the *A. thaliana* genome. An annotation utilizing *A. thaliana* proteins and transcripts (48,359 transcripts) was utilized as positive control.

Benchmarking Universal Single‐Copy Orthologs (BUSCO) (Simão et al., 2015) was run on predicted transcriptomes from the various annotations using the viridiplantae_odb10 database and compared to the *A. thaliana* published transcriptome. *PSAURON v.1.0.6* (Sommer et al., 2025) (where PSAURON is protein structure audit‐oriented evaluation) was used to evaluate the quality of proteins annotated. Iso‐seq transcriptomic reads from different tissues and stresses of *A. thaliana* were downloaded from the Sequence Read Archive (Table S1). Whole‐genome protein and transcript sequences from the different species were downloaded from NCBI (Table S2).

### 2.4 Validation of the IWGC annotation pipeline using crop species

Genomes from *Zea mays* (Poaceae) (GCF_902167145.1), *Sorghum bicolor* (Poaceae) (GCA_000003195.3), *G. max* (Fabaceae) (GCF_000004515.6), *Solanum lycopersicum* (Solanaceae) (GWHFILF00000000.3), and *Solanum tuberosum* (GCA_020169585.1) (Solanaceae) were reannotated using the IWGC pipeline. Iso‐seq reads of the same species, but proteins from four botanically close species, were utilized to annotate the genomes. The number of Iso‐seq reads and the protein source used as input to the pipeline are described in Table S3. The number of genes annotated was counted at the end of the pipeline and compared to the number of genes in the publicly available annotation file of each genome.

## 3 RESULTS

**FIGURE 2:**
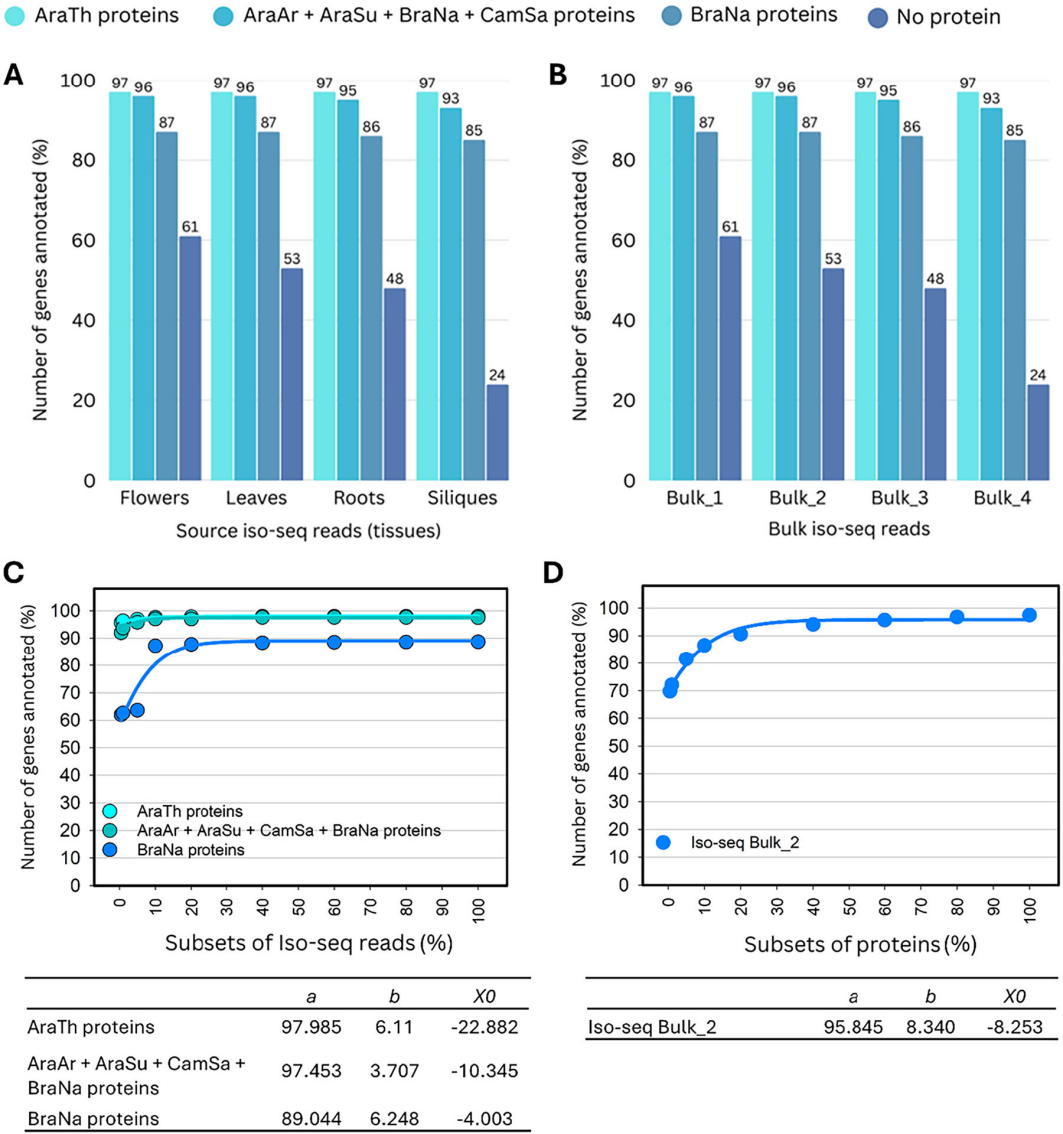
(A) Percentage of *Arabidopsis thaliana* (AraTh) genes annotated utilizing isoform sequencing (Iso‐seq) from different tissues (flowers, leaves, roots, and siliques) combined with proteins from only *A. thaliana*, combination of *A. arenosa* (AraAr) + *A. suecica* (AraSu) + *Brassica napus* (BraNa) + *Camelina sativa* (CamSa), only *B. napus*, or no proteins. (B) Percentage of *A. thaliana* genes annotated utilizing Iso‐seq from the combination (bulk) of different tissues, abiotic, and biotic stresses and combined with proteins from only *A. thaliana*, combination of *A. arenosa* + *A. suecica* + *B. napus* + *C. sativa*, only *B. napus*, or no proteins. (C) Percentage of *A. thaliana* genes annotated utilizing subsets from Bulk_2 of Iso‐seq (leaves + flowers + roots + siliques + seedlings) combined with proteins from only *A. thaliana*, a combination of *A. arenosa* + *A. suecica* + *B. napus* + *C. sativa*, or only *B. napus*, and fitted to a three‐parameter sigmoidal curve. Equation parameters are provided in the table below the image. (D) Percentage of *A. thaliana* genes annotated utilizing Bulk_2 of Iso‐seq (leaves + flowers + roots + siliques + seedlings) and subsets of the proteins from the combination of *A. arenosa + A. suecica + B. napus + C. sativa* and fitted to a three‐parameter sigmoidal curve. Equation parameters are provided in the table below the image.

The use of Iso‐seq reads generated from different tissues or the combination with biotic and abiotic stresses had little effect on the total number of genes annotated using this pipeline (Figure 2A,B); however, the relatedness of the species used as the source of proteins utilized in the gene model prediction had a relatively large effect. Utilizing proteins from *A. thaliana* or from the combination of *A. arenosa* + *A. suecica* + *B. napus* + *C. sativa* as input for the gene model prediction annotated 93%–98% of the total 27,561 genes annotated in the most recent *A. thaliana* genome paper (Hou et al., 2022). However, if only proteins from *B. napus* were used, the range was 85%–89%. If only Iso‐seq data were used (i.e., no protein information), only 24%–67% of total expected genes were predicted.

The effect of Iso‐seq reads quantity was investigated by subsetting the number of reads and keeping the number of proteins constant. The utilization of a small or high number of Iso‐seq reads did not greatly affect the total number of genes annotated when using proteins from the same species, *A. thaliana* (Figure 2C). For example, the Iso‐seq subset of 0.5%, containing 98,528 reads, annotated 96% of genes, while the 100% subset, containing 19,735,858 reads, annotated 98%. The utilization of a combination of proteins from different species, *A. arenosa* + *A. suecica* + *B. napus* + *C. sativa*, decreased the number of genes annotated when utilizing the smaller number of Iso‐seq reads, with 92% and 94% of the total genes annotated when using 0.5% and 1% Iso‐seq reads subsets, respectively (Figure 2C). When utilizing Iso‐seq subsets from 20% to 100%, there was only a difference of 0.4% in the number of genes annotated between utilizing *A. thaliana* proteins and the combination of *A. arenosa* + *A. suecica* + *B. napus* + *C. sativa* proteins (Figure 2C). When only *B. napus* proteins were utilized, an *A. thaliana* taxonomically more distant species than *A. arenosa*, *A. suecica*, and *C. sativa*, the number of annotated genes ranged from 62% to 89% across the Iso‐seq reads subsets (Figure 2C). To evaluate the effect of protein amount, we utilized a constant high number of Iso‐seq reads (Bulk_2: 19,735,858 reads), but different protein subsets affected the total number of genes annotated. The 100% subset of proteins refers to the combination of *A. arenosa* + *A. suecica* + *B. napus* + *C. sativa* proteins, totaling 314,397 proteins. The number of proteins directly correlated to annotation completeness, with the fewest proteins resulting in the fewest gene model calls (Figure 2D). The protein subset of 10%, containing 31,599 proteins, annotated 86.5% of genes, while the protein subset of 100% annotated 97.5% of genes, highlighting the importance of utilizing a high number of proteins from closely related species as evidence in the gene model prediction (Figure 2D).

**FIGURE 3:**
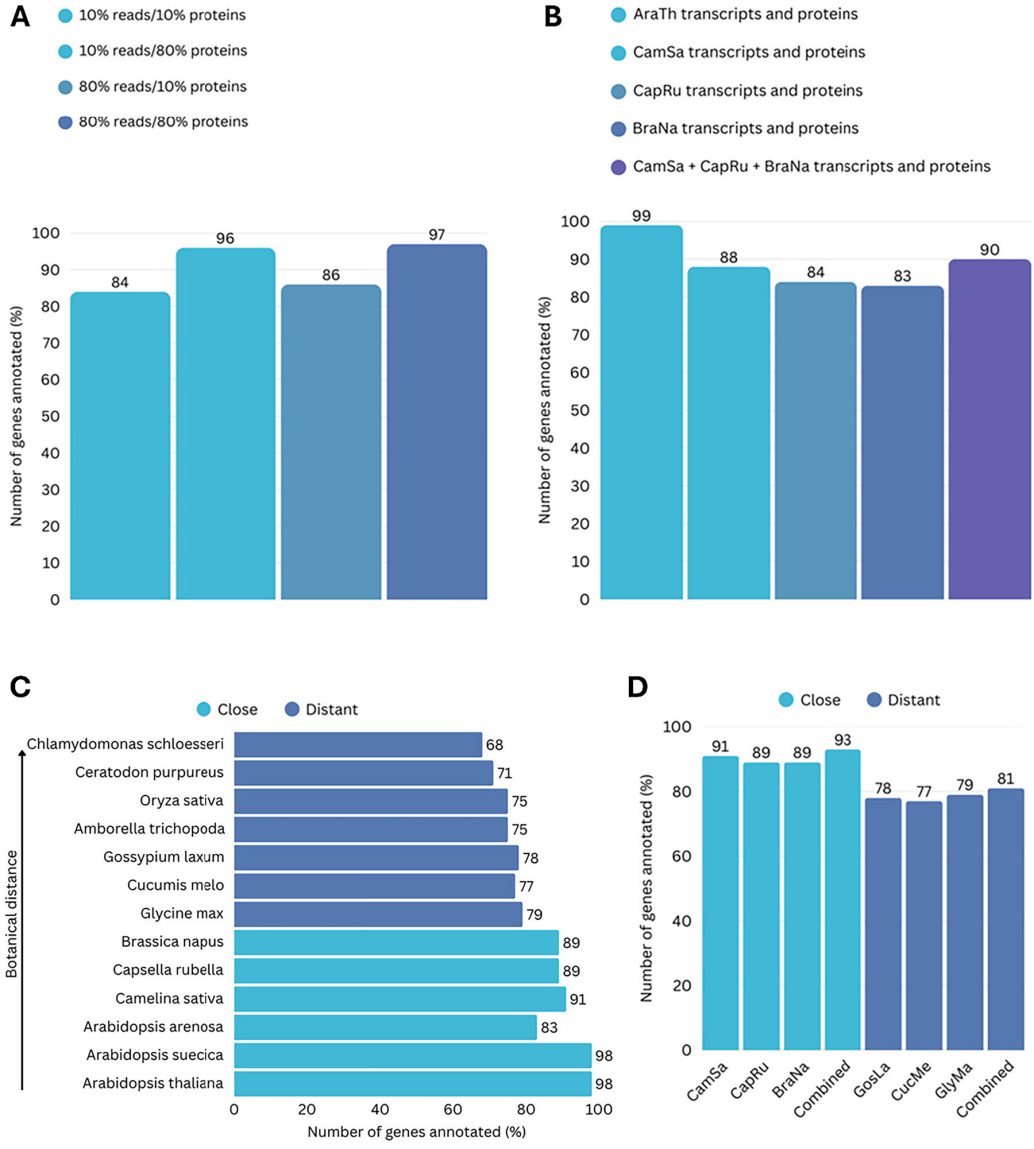
(A) Percentage of *Arabidopsis thaliana* (AraTh) genes annotated utilizing the combination of 10% and 80% subsets of isoform sequencing (Iso‐seq) and proteins. The number of reads and proteins of each percentage is described in Table 2. (B) Percentage of *A. thaliana* genes annotated utilizing transcripts and proteins from *A. thaliana*, *Camelina sativa* (CamSa), *Capsella rubella* (CapRu), or *Brassica napus* (BraNa), and the combination of *C. sativa* + *C. rubella* + *B. napus*. (C) Percentage of *A. thaliana* genes annotated utilizing proteins from different species closely and distantly related to *A. thaliana*. The Iso‐seq reads source was Bulk_2 (19,735,858 reads). (D) Detailed results from subpart (C) focusing on the percentage of *A. thaliana* genes annotated utilizing three closely related species (*C. sativa*, *C. rubella*, and *B. napus*) and three distantly related species (*Gossypium laxum* [GosLa], *Cucumis melo* [CucMe], and *Glycine max* [GlyMa]) *A. thaliana*, separated and combined by botanical proximity

The combination of Iso‐seq reads subset of 10% (containing 1,974,803 reads) + proteins subset of 10% (containing 31,599 proteins) annotated 84% of genes; the combination of Iso‐seq subset of 80% (containing 115,788,610 reads) + proteins subset of 80% (containing 251,356 proteins) annotated 97% (Figure 3A). However, keeping the Iso‐seq subset at 10% and the protein subset at 80% was able to increase to 96% the number of genes, while increasing the Iso‐seq subset to 80% combined with the protein subset of 10% annotated 86% of genes (Figure 3A). The BUSCO score analysis shows that the Iso‐seq subset of 10% + proteins subset of 80% had a completeness (C) score of 96%, while the opposite Iso‐seq subset of 80% + proteins subset of 10% had a C score of 89% (Table 4). These results show that the number of proteins has a relatively larger impact than the number of Iso‐seq reads in the gene model prediction step, or it could be that the number of Iso‐seq reads needed to achieve basal annotation levels is lower than was tested here.

**TABLE 4:** Benchmarking Universal Single‑Copy Orthologs (BUSCO) scores of *Arabidopsis thaliana* genome and transcriptomes from different annotation scenarios.

	Transcriptome BUSCO scores (viridiplantae)			PSAURON scores
Species	C (%)	F (%)	M (%)	
*A. thaliana* transcriptome (Hou et al., 2022)	99.3	0.0	0.7	–
*A. thaliana* proteome (Hou et al., 2022)	–	–	–	96.2
				
Reads subset 10% + proteins subset 10%	92.0	2.8	5.2	97.2
Reads subset 10% + proteins subset 80%	96.2	2.8	1.0	95.5
Reads subset 80% + proteins subset 10%	89.2	6.6	4.2	97.0
Reads subset 80% + proteins subset 80%	91.3	7.1	1.6	95.3
				
Bulk_2 Iso‐seq reads + *A. thaliana* proteins	92.2	5.4	2.4	95.1
Bulk_2 Iso‐seq reads + *C. sativa* proteins	91.8	5.9	2.3	96.7
Bulk_2 Iso‐seq reads + *C. rubella* proteins	92.3	5.4	2.3	96.4
Bulk_2 Iso‐seq reads + *B. napus* proteins	92.5	5.4	2.1	96.9
Bulk_2 Iso‐seq reads + *G. laxum* proteins	90.3	5.6	4.1	97.6
Bulk_2 Iso‐seq reads + *C. melo* proteins	91.7	5.4	2.9	97.7
Bulk_2 Iso‐seq reads + *G. max* proteins	91.5	5.9	2.6	97.6
Bulk_2 Iso‐seq reads + three closely related species	92.2	5.6	2.2	96.4
Bulk_2 Iso‐seq reads + three distant related species	92.2	5.9	1.9	97.3

*Note*: C: complete; F: fragmented; M: missing.

Abbreviations: Iso‐seq, isoform sequencing; PSAURON, protein structure audit oriented evaluation.

In a simulated scenario where no Iso‐seq reads were used to annotate the genome of *A. thaliana*, the utilization of both transcripts and proteins from closely related species annotated a reasonable number of genes (Figure 3B). In this simulation, the transcripts are the annotated transcripts linked to the genome of each species. *C. sativa*, *C. rubella*, or *B. napus* transcripts and proteins annotated 88%, 84%, and 83% of genes, respectively. The combination of transcripts and proteins from all three species increased annotation to 90% of genes (Figure 3B). As a control, the utilization of transcripts and proteins from *A. thaliana* annotated 99% of genes.

Utilizing the three closely related species, *C. sativa*, *C. rubella*, and *B. napus* annotated 91%, 89%, and 89% of genes, respectively, while utilizing the three more distantly related species *G. laxum*, *C. melo*, or *G. max* annotated 78%, 77%, and 79%, respectively (Figure 3C,D). The combination of the proteins from these three closely related species annotated 93% of the genes, while the combination of proteins from the three more distant species annotated 81% (Figure 3D). The combination of proteins from different species, rather than individual species, increased the number of genes annotated by at least 2%. It is important to mention that the different species have different numbers of proteins (Table 3), and as shown before, the protein amount affects the total number of genes annotated (Figure 2D). However, the combination of the number of proteins × botanical distance does not follow a pattern. *A. thaliana*’s closest relative *A. suecica* has 63,469 proteins and annotated 97.5% of genes, while the more distant relative *B. napus* has 120,350 proteins and annotated 88.7% of genes; on the other hand, *B. napus* annotated 5% more genes than *A. arenosa* (which has 23,097 proteins) (Figure 3C). Overall, when utilizing proteins from close relatives of *A. thaliana*, more genes were annotated, while more proteins from distantly related species resulted in fewer genes annotated.

The BUSCO scores of the transcriptomes annotated when utilizing proteins from different species ranged from 90% to 92% for completeness (C) (Table 4). The completeness of the *A. thaliana* transcriptome from the original article (Hou et al., 2022) is 99%, while the transcriptome generated with the IWGC annotation pipeline utilizing *A. thaliana* Iso‐seq reads and proteins was 92% (Table 4). The BUSCO scores for the combinations of reads and protein subset (10% and 80% each) ranged from 89% to 96% completeness, but it was not possible to associate the higher completeness with a higher number of proteins or Iso‐seq reads (Table 4). The quality of the proteins annotated was high in all scenarios evaluated according to the PSAURON scores, with >95% of the proteins annotated being in‐frame (Table 4).

The reannotation of crop species from different botanical families utilizing the IWGC pipeline resulted in a very good number of genes annotated, but slightly lower than observed for *A. thaliana*, except for *S. tuberosum*. The annotation of *Z. mays* and *S. bicolor*, two Poaceae, resulted in 87.2% and 90.2% of genes annotated, respectively, when compared to the annotation published along with the genome utilized (Table 5). The annotation of the Fabaceae *G. max* resulted in 94.6% of genes being annotated, whereas the Solanaceae *S. lycopersicum* and *S. tuberosum* were in 89.7% and 100.5% compared to their publicly available annotation (Table 5).

**TABLE 5:** Number of genes annotated using the International Weed Genomics Consortium (IWGC) pipeline compared to the publicly available annotation of four crop species genomes.

		Number of genes		
Species	Family	Original annotation	IWGC annotation	Genes annotated (%)
*Zea mays*	Poaceae	49,902	43,531	87.2
*Sorghum bicolor*	Poaceae	34,118	30,769	90.2
*Glycine max*	Fabaceae	52,872	50,018	94.6
*Solanum lycopersicum*	Solanaceae	36,006	32,316	89.7
*Solanum tuberosum*	Solanaceae	37,965	38,139	100.5

## 4 DISCUSSION

We present the IWGC genome annotation pipeline, a compilation of industry‐standard gene annotation tools wrapped together into a single containerized application for efficient and easy installation and use, even for non‐computational biologists. This robust pipeline has now been used to annotate gene models in many diverse non‐model plant species (Gomes et al., 2025; J. Lemas, Ņečajeva, et al., 2025; J. M. Lemas, Patterson, et al., 2025; Montgomery et al., 2024; Raiyemo, Cutti, et al., 2025; Raiyemo, Montgomery, et al., 2025) and accommodates variation in ploidy and genome size. Here, we assess the sensitivity of this pipeline to the types, amount, and quality of data inputs in terms of number of genes predicted versus what should be predicted. We show that, given the highest quality inputs available, our automated pipeline annotates nearly as many genes as the more in‐depth, manually curated approaches used in the *A. thaliana* genome paper (Hou et al., 2022).

While full‐length transcript sequencing has been used to better understand splice variation and discover new protein isoforms (Miller et al., 2022), it may also overcome the limitations faced with short‐reads in predicting gene structure (Paniagua et al., 2025). The amount of Iso‐seq data only notably improved our pipeline's ability to predict gene models when proteins from closely related species were not available. By default, *MAKER‐P* annotates genes that are supported by evidence and ab initio gene prediction that encode a *Pfam* domain (Campbell, Holt, et al., 2014). Ab initio methods use statistical approach from the genome sequence to generate predictions (Baker et al., 2023; Holst et al., 2025). We should note that ab initio gene prediction methods were trained using *A. thaliana* data (Stanke et al., 2006), and this training likely contributes to establishing a baseline level of annotation. Long reads, full length, and high quality are more important for accurate transcript model prediction than utilizing increased read depth (Paniagua et al., 2025; Pardo‐Palacios et al., 2024). Conversely, the pipeline is quite sensitive to the quantity and relatedness of reference proteins set from related species, even when Iso‐seq sequencing from multiple tissues is used. While reference protein sets from closely related species are not always available, especially for researchers of non‐model species like weeds, combining the gene sets of multiple more distantly related species increased the pipeline's ability to predict gene models. However, it has to be considered that using information from other related species may result in propagation of incorrect annotation (Scalzitti et al., 2020). Interestingly, data input quantity and relatedness did not have a notable effect on the prediction of BUSCO gene models with the inclusion of more isoform sequence data reducing the number of complete models when species‐specific gene sets were used. These results highlight the importance of using quality protein sets from closely related species to produce the best annotation of gene models in new species. While annotations produced through our pipeline are generally accurate, researchers should consider gathering transcript sequence information from genotypes of interest to best understand expression and splicing patterns underlying traits of interest.

The IWGC annotation pipeline is an evidence‐driven genome annotation pipeline combining extrinsic evidence and ab initio algorithms, offering a valuable compromise of a small reduction in prediction accuracy for the reduction in inputs and expertise required for more manually curated annotation methods. Previous work comparing automated and manually curated gene annotations showed that manual curation changed automated annotations very little, and therefore in silico predictions are reliable (Wilbrandt et al., 2019). In non‐model systems, such as weed species, without the resources and researchers available for expensive and time‐consuming manual curation, in silico prediction methods offer a valuable compromise of a small reduction in accuracy compared to the massive reduction in required input resources. This genome annotation pipeline has been utilized to annotate the weedy genomes from diploid *A. palmeri*, *Amaranthus hybridus*, *Amaranthus retroflexus* (Raiyemo, Montgomery, et al., 2025), *A. tuberculatus* (Raiyemo, Cutti, et al., 2025), *Apera spica‐venti* (J. Lemas, Ņečajeva, et al., 2025), allotetraploid *Salsola tragus* (J. M. Lemas, Patterson, et al., 2025), and hexaploids *Conyza sumatrensis* and *Conyza bonariensis* (Gomes et al., 2025). The annotation of these non‐model species was found to be complete, with transcriptome BUSCO scores of 96% for *C. sumatrensis* and *C. bonariensis*, while for *S. tragus* the transcriptome BUSCO score was 88%. According to our results, it is possible to reduce project costs by reducing the amount of Iso‐seq reads sequenced and increasing the quality and quantity of proteins as input in the pipeline, with a small or no reduction in the total number of genes annotated.

The annotation of crop species genomes from *Z. mays*, *S. bicolor* (Poaceae), *G. max* (Fabaceae), and *S. lycopersicum*, which are from different botanical families than *A. thaliana* (Brassicaceae), resulted in nearly 90% of genes annotated. However, this number is slightly lower than the number of *A. thaliana* genes annotated (96%). The *S. tuberosum* annotation outperformed, annotating the same number of genes as the publicly available annotation. These results show that there is a variability of annotation efficacy across botanical families and within the same botanical family. The number of Iso‐seq reads and proteins was not a limitation to annotate the crops’ genomes, because many reads and Iso‐seqs were utilized. The *A. thaliana* annotation performed better likely because the ab initio gene prediction methods were trained using *A. thaliana* data.

Deep learning and other machine learning approaches continue to rapidly improve as new architectures, creative approaches like transfer learning, and more omics data are produced. The expansion of the field of artificial intelligence has led to the development of new tools to annotate genomic features such as genes, transposable elements, and cis‐regulatory elements (Peleke et al., 2024; Stiehler et al., 2021; Yan et al., 2020). However, current pre‐trained deep learning models are frequently unacceptable for accurate annotation, especially in non‐model systems, and training these models and validating their predictions requires expensive data, which often needs to be species‐specific. Anecdotally, our experience with this first wave of artificial intelligence (AI)‐only‐based annotation tools is that they often make mistakes in exon‐exon junctions, produce numerous small introns and/or exons that are fallacious, and improperly split or join nearby genes. As new AI tools are developed and tested, their integration may allow our pipeline to become more powerful and robust to the quality and relatedness of data inputs.

The IWGC annotation pipeline was efficient in reannotating the model species *A. thaliana*, and we learned that increasing the number of proteins utilized as evidence in the gene model prediction step has a higher effect on increasing the number of genes annotated when compared to increasing the number of Iso‐seq reads. In addition to the number of proteins, the utilization of proteins from closely related species is also critical for better annotation. This pipeline has been used to annotate non‐model weed species genomes from IWGC and is provided for use as an open‐source tool.

## AUTHOR CONTRIBUTIONS

**Luan Cutti**: Data curation; formal analysis; investigation; methodology; validation; writing—original draft. **Daniel Fernando da Silva Filho**: Formal analysis; methodology; software; writing—review and editing. **Geisson Edwin Guadir Lara**: Formal analysis; methodology; software; writing—review and editing**. Jessica Matheson**: Investigation; methodology; writing—review and editing. **Nicholas A. Johnson**: Formal analysis; methodology; writing—original draft**. Jacob Montgomery**: Writing—original draft. **Nathan Hall**: Methodology; software. **Brent Murphy**: Funding acquisition; project administration; writing—review and editing. **Todd A. Gaines**: Conceptualization; funding acquisition; project administration; supervision; writing—original draft. **Eric L. Patterson**: Conceptualization; funding acquisition; investigation; methodology; project administration; software; supervision; writing—original draft.

## CONFLICT OF INTEREST STATEMENT

The authors declare no conflicts of interest.

The whole pipeline and scripts are available on the GitHub page:

Structural annotation: https://github.com/danifilho/IWGC_Structural

Functional annotation: https://github.com/danifilho/IWGC_Funcional

Pipeline explanation: https://github.com/PattersonWeedLab/IWGC_annotation_pipeline

## Supporting information



Table S1. SRA IDs of Iso‐seq reads utilized to reannotate *Arabidopsis thaliana* genome
Table S2. NCBI IDs of proteins and transcripts utilized to reannotate *Arabidopsis thaliana* genome
Table S3. NCBI IDs of proteins and SRA IDs of Iso‐seq reads utilized to reannotate crop genomes
Figure S1. Phylogenetic tree of species used as protein sources for the annotation of the Arabidopsis thaliana genome

## Data Availability

The dataset supporting the conclusions of this article is included within the article (and its additional file).
