# Supplementary material for: Validation of the International Weed Genomics Consortium genome annotation pipeline through reannotation of the model species Arabidopsis thaliana
Source: Plant Genome. 2026 Jun 30;19(3):e70270. doi: 10.1002/tpg2.70270 (PMC13316137; doi:10.1002/tpg2.70270)
Supplement: Supplementary file 1 [file TPG2-19-e70270-s001.docx]

**SUPPLEMENTARY MATERIAL**

**Validation of the International Weed Genomics Consortium genome annotation pipeline through reannotation of the model species *Arabidopsis thaliana***

Luan Cutti^1^, Daniel Fernando da Silva Filho^2^, Geisson Edwin Guadir Lara^3^, Jessica Matheson^4^, Nicholas A. Johnson^5^, Jacob Montgomery^6^, Nathan Hall^7^, Brent Murphy^8^, Todd A. Gaines^9^, Eric L. Patterson^10*^

^1^ Department of Plant, Soil, and Microbial Sciences, Michigan State University, East Lansing, MI, USA.

^2^ Department of Biological Sciences, São Paulo State University, Bauru, SP, Brazil.

^3^ Department of Plant, Soil, and Microbial Sciences, Michigan State University, East Lansing, MI, USA.

^4^ Department of Biology, University of Massachusetts Amherst, Amherst, MA, USA.

^5^ Department of Plant, Soil, and Microbial Sciences, Michigan State University, East Lansing, MI, USA.

^6^ Department of Plant, Soil, and Microbial Sciences, Michigan State University, East Lansing, MI, USA.

^7^ Department of Plant, Soil, and Microbial Sciences, Michigan State University, East Lansing, MI, USA.

^8^ BASF SE, Agricultural Research Station, Limburgerhof, Germany.

^9^ Department of Agricultural Biology, Colorado State University, Fort Collins, CO, USA.

^10^ Department of Plant, Soil, and Microbial Sciences, Michigan State University, East Lansing, MI, USA.

^*^Corresponding Author: Eric L. Patterson. Department of Plant, Soil, and Microbial Sciences, Michigan State University, East Lansing, MI, 48824, USA. Email: patte543@msu.edu

**Docker images**

In this project, a set of Docker images for both structural and functional annotation phases were prepared. Some images were custom-built to include specific versions of tools and dependencies tailored to the pipeline, while others were obtained directly from Docker Hub.

●	Custom-Built Docker Images:

○	[Maker v3.01]: Built to run isolated with all its necessary dependencies for structural annotation and interacts with custom scripts. MAKER generates individual chromosome annotations, which are combined into a global GFF file, keeping only selected feature types;

○	[Custom python 3.0]: Includes some packages such as gffread v0.12, used for protein extraction, indexation, and filter based on a length threshold;

○	[sigtarp]: Used for predicted signal peptides and targeting signals in the functional annotation phase.

●	Docker Hub Images:

○	[dfam/tetools]: Retrieved from Docker Hub to provide Repeat Modeler and Repeat Masker for identifying repetitive DNA elements using RepeatModeler and masks them with RepeatMasker, in the structural annotation phase.

○	[pegi3s/bedtools]: Used for bedtools, that then soft-masks the genome sequences in the structural annotation phase.

○	[greensii/isoseq3]: Used for accessing pbmm2 and isoseq3. Here, Isoseq reads are aligned to the masked genome, followed by collapsing redundant transcripts in the structural annotation phase.

○	[dbest/samtools:v1.19.2]: Retrieval of samtools, used in custom scripts and in different parts of each phase.

○	[agat]: Used for filtering the GFF file to keep the longest isoform in the functional annotation phase.

○	[hmmer3]: Used for identifying protein domains using Pfam-A in the functional annotation phase.

○	[iprscan]: Used for performing functional annotation in the functional annotation phase.

○	[mmseqs2]: Used for searching protein sequences against specified databases in the functional annotation phase.

○	[multiloc2]: Used for predicted subcellular localization in the functional annotation phase.

Each Docker image was documented with the exact version numbers and the source (custom-built or Docker Hub).

**Table S1.** SRA IDs of Iso-seq reads utilized to reannotate *Arabidopsis thaliana* genome

|  | Iso-seq SRA IDs |
| --- | --- |
| Leaves | SRR14584396 |
| Flowers | SRR14584397 |
| Roots | SRR23291388, SRR15498066 |
| Siliques | SRR23291386, SRR15498064 |
| Seedlings | SRR14584394 |
| Cold | SRR23291390, SRR15498068 |
| Heat | SRR23291399, SRR15498077 |
| Flooding | SRR23291378, SRR15498056 |
| *Botrytis cinerea* | SRR23291397, SRR15498075 |
| *Pseudomonas syringae* | SRR15498071, SRR23291393 |
| *Hyaloperonospora arabidopsidis* | SRR23291394, SRR15498072 |

**Table S2.** NCBI IDs of proteins and transcripts utilized to reannotate *Arabidopsis thaliana* genome

|  | Proteins and/or transcripts |
| --- | --- |
| *Arabidopsis thaliana* | Hou et al., 2022 |
| *Arabidopsis suecica* | GCA_019202805.1 |
| *Arabidopsis arenosa* | GCA_905216605.1 |
| *Camelina sativa* | GCF_000633955.1 |
| *Capsella rubella* | GCF_000375325.1 |
| *Brassica napus* | GCF_020379485.1 |
| *Glycine max* | GCF_000004515.6 |
| *Cucumis melo* | GCF_025177605.1 |
| *Gossypium laxum* | GCA_013511315.1 |
| *Amborella trichopoda* | GCF_000471905.2 |
| *Oryza sativa* | GCF_001433935.1 |
| *Ceratodon purpureus* | GCA_014871385.1 |
| *Chlamydomonas schloesseri* | GCA_016834595.1 |

**Table S3.** NCBI IDs of proteins and SRA IDs of Iso-seq reads utilized to reannotate *Zea mays*, *Sorghum bicolor* (Poaceae), *Glycine max* (Fabaceae), *Solanum lycopersicum*, and *Solanum tuberosum* (Solanaceae) genomes

|  | Iso-seq reads source | | Proteins source | | |
| --- | --- | --- | --- | --- | --- |
| Crop species reannotated | NCBI SRA ID | Total number of reads | Species | Total number of proteins | NCBI ID |
| *Zea mays* | SRR23046702 | 6765236 | *Setaria italica* | 207428 | GCA_000263155.2 |
|  |  |  | *Digitaria exilis* |  | GCA_015342445.1 |
|  |  |  | *Panicum miliaceum* |  | GCA_003046395.2 |
|  |  |  | *Sorghum bicolor* |  | GWHEQHX00000000 |
| *Sorghum bicolor* | SRR34258085 | 6262038 | *Setaria italica* | 218248 | GCA_000263155.2 |
|  | SRR34258119 |  | *Digitaria exilis* |  | GCA_015342445.1 |
|  | SRR34258120 |  | *Panicum miliaceum* |  | GCA_003046395.2 |
|  |  |  | *Zea mays* |  | GCA_902167145.1 |
| *Glycine max* | SRR10305130 | 557147 | *Mucuna pruriens* | 171252 | GCA_003370565.1 |
|  | SRR10309858 |  | *Phaseolus vulgaris* |  | GCA_000499845.2 |
|  | SRR10309859 |  | *Pueraria montana* |  | GWHBEIM00000000 |
|  | SRR10309860 |  | *Vigna radiata* |  | GWHEQVC00000000 |
|  | SRR10309861 |  |  |  |  |
|  | SRR10309862 |  |  |  |  |
|  | SRR10309863 |  |  |  |  |
|  | SRR10309864 |  |  |  |  |
|  | SRR10309865 |  |  |  |  |
|  | SRR10309866 |  |  |  |  |
|  | SRR10309867 |  |  |  |  |
|  | SRR10309868 |  |  |  |  |
|  | SRR10309869 |  |  |  |  |
|  | SRR10309870 |  |  |  |  |
|  | SRR10309871 |  |  |  |  |
|  | SRR10309872 |  |  |  |  |
|  | SRR10309873 |  |  |  |  |
|  | SRR10309874 |  |  |  |  |
|  | SRR10309875 |  |  |  |  |
|  | SRR10309876 |  |  |  |  |
|  | SRR10309877 |  |  |  |  |
|  | SRR10309878 |  |  |  |  |
|  | SRR10309879 |  |  |  |  |
| *Solanum lycopersicum* | SRR24295332 | 1507736 | *Solanum tuberosum* | 181534 | GCA_020169535.1 |
|  | SRR24295336 |  | *Capsicum annuum* |  | GCA_011745845.1 |
|  |  |  | *Nicotiana tabacum* |  | GWHGEXH00000000.1 |
|  |  |  | *Solanum torvum* |  | GWHCAXF00000000 |
| *Solanum tuberosum* | *SRR14298411* | 3976347 | *Solanum lycopersicum* | 180523 | GWHFILF00000000.3 |
|  | *SRR14298438* |  | *Capsicum annuum* |  | GCA_011745845.1 |
|  | *SRR14298440* |  | *Nicotiana tabacum* |  | GWHGEXH00000000.1 |
|  |  |  | *Solanum torvum* |  | GWHCAXF00000000 |

**Figure S1**. Phylogenetic tree of species used as protein sources for the annotation of the *Arabidopsis thaliana* genome: *Arabidopsis thaliana* (AraTh), *Arabidopsis suecica* (AraSu), *Arabidopsis arenosa* (AraAr), *Camelina sativa* (CamSa), *Capsella rubella* (CapRu), *Brassica napus* (BraNa), *Glycine max* (GlyMa), *Cucumis melo* (CucMe), *Gossypium laxum* (GosLa), *Amborella trichopoda* (AmbTr), *Oryza sativa* (OrySa), *Ceratodon purpureus* (CerPu), *Chlamydomonas schloesseri* (ChlSc).
