# Supplementary material for: Validation of the International Weed Genomics Consortium genome annotation pipeline through reannotation of the model species Arabidopsis thaliana
Source: Plant Genome. 2026 Jun 30;19(3):e70270. doi: 10.1002/tpg2.70270 (PMC13316137; doi:10.1002/tpg2.70270)
Supplement: Supplementary file 1 — Table S1. SRA IDs of Iso‐seq reads utilized to reannotate Arabidopsis thaliana genome Table S2. NCBI IDs of proteins and transcripts utilized to reannotate Arabidopsis thaliana genome Table S3. NCBI IDs of proteins and SRA IDs of Iso‐seq reads utilized to reannotate crop genomes Figure S1. Phylogenetic tree of species used as protein sources for the annotation of the Arabidopsis thaliana genome [file TPG2-19-e70270-s001.docx]

**SUPPLEMENTARY MATERIAL**

**Validation of the International Weed Genomics Consortium genome annotation pipeline through reannotation of the model species *Arabidopsis thaliana***

Luan Cutti^1^, Daniel Fernando da Silva Filho^2^, Geisson Edwin Guadir Lara^3^, Jessica Matheson^4^, Nicholas A. Johnson^5^, Jacob Montgomery^6^, Nathan Hall^7^, Brent Murphy^8^, Todd A. Gaines^9^, Eric L. Patterson^10*^

^1^ Department of Plant, Soil, and Microbial Sciences, Michigan State University, East Lansing, MI, USA.

^2^ Department of Biological Sciences, São Paulo State University, Bauru, SP, Brazil.

^3^ Department of Plant, Soil, and Microbial Sciences, Michigan State University, East Lansing, MI, USA.

^4^ Department of Biology, University of Massachusetts Amherst, Amherst, MA, USA.

^5^ Department of Plant, Soil, and Microbial Sciences, Michigan State University, East Lansing, MI, USA.

^6^ Department of Plant, Soil, and Microbial Sciences, Michigan State University, East Lansing, MI, USA.

^7^ Department of Plant, Soil, and Microbial Sciences, Michigan State University, East Lansing, MI, USA.

^8^ BASF SE, Agricultural Research Station, Limburgerhof, Germany.

^9^ Department of Agricultural Biology, Colorado State University, Fort Collins, CO, USA.

^10^ Department of Plant, Soil, and Microbial Sciences, Michigan State University, East Lansing, MI, USA.

^*^Corresponding Author: Eric L. Patterson. Department of Plant, Soil, and Microbial Sciences, Michigan State University, East Lansing, MI, 48824, USA. Email: patte543@msu.edu

**Docker images**

In this project, a set of Docker images for both structural and functional annotation phases were prepared. Some images were custom-built to include specific versions of tools and dependencies tailored to the pipeline, while others were obtained directly from Docker Hub.

● Custom-Built Docker Images:

○ [Maker v3.01]: Built to run isolated with all its necessary dependencies for structural annotation and interacts with custom scripts. MAKER generates individual chromosome annotations, which are combined into a global GFF file, keeping only selected feature types;

○ [Custom python 3.0]: Includes some packages such as gffread v0.12, used for protein extraction, indexation, and filter based on a length threshold;

○ [sigtarp]: Used for predicted signal peptides and targeting signals in the functional annotation phase.

● Docker Hub Images:

○ [dfam/tetools]: Retrieved from Docker Hub to provide Repeat Modeler and Repeat Masker for identifying repetitive DNA elements using RepeatModeler and masks them with RepeatMasker, in the structural annotation phase.

○ [pegi3s/bedtools]: Used for bedtools, that then soft-masks the genome sequences in the structural annotation phase.

○ [greensii/isoseq3]: Used for accessing pbmm2 and isoseq3. Here, Isoseq reads are aligned to the masked genome, followed by collapsing redundant transcripts in the structural annotation phase.

○ [dbest/samtools:v1.19.2]: Retrieval of samtools, used in custom scripts and in different parts of each phase.

○ [agat]: Used for filtering the GFF file to keep the longest isoform in the functional annotation phase.

○ [hmmer3]: Used for identifying protein domains using Pfam-A in the functional annotation phase.

○ [iprscan]: Used for performing functional annotation in the functional annotation phase.

○ [mmseqs2]: Used for searching protein sequences against specified databases in the functional annotation phase.

○ [multiloc2]: Used for predicted subcellular localization in the functional annotation phase.

Each Docker image was documented with the exact version numbers and the source (custom-built or Docker Hub).

**Table S1.** SRA IDs of Iso-seq reads utilized to reannotate *Arabidopsis thaliana* genome

|  | Iso-seq SRA IDs |
| --- | --- |
| Leaves | SRR14584396 |
| Flowers | SRR14584397 |
| Roots | SRR23291388, SRR15498066 |
| Siliques | SRR23291386, SRR15498064 |
| Seedlings | SRR14584394 |
| Cold | SRR23291390, SRR15498068 |
| Heat | SRR23291399, SRR15498077 |
| Flooding | SRR23291378, SRR15498056 |
| *Botrytis cinerea* | SRR23291397, SRR15498075 |
| *Pseudomonas syringae* | SRR15498071, SRR23291393 |
| *Hyaloperonospora arabidopsidis* | SRR23291394, SRR15498072 |

**Table S2.** NCBI IDs of proteins and transcripts utilized to reannotate *Arabidopsis thaliana* genome

|  | Proteins and/or transcripts |
| --- | --- |
| *Arabidopsis thaliana* | Hou et al., 2022 |
| *Arabidopsis suecica* | GCA_019202805.1 |
| *Arabidopsis arenosa* | GCA_905216605.1 |
| *Camelina sativa* | GCF_000633955.1 |
| *Capsella rubella* | GCF_000375325.1 |
| *Brassica napus* | GCF_020379485.1 |
| *Glycine max* | GCF_000004515.6 |
| *Cucumis melo* | GCF_025177605.1 |
| *Gossypium laxum* | GCA_013511315.1 |
| *Amborella trichopoda* | GCF_000471905.2 |
| *Oryza sativa* | GCF_001433935.1 |
| *Ceratodon purpureus* | GCA_014871385.1 |
| *Chlamydomonas schloesseri* | GCA_016834595.1 |

**Table S3.** NCBI IDs of proteins and SRA IDs of Iso-seq reads utilized to reannotate *Zea mays*, *Sorghum bicolor* (Poaceae), *Glycine max* (Fabaceae), *Solanum lycopersicum*, and *Solanum tuberosum* (Solanaceae) genomes

|  | Iso-seq reads source | | Proteins source | | |
| --- | --- | --- | --- | --- | --- |
| Crop species reannotated | NCBI SRA ID | Total number of reads | Species | Total number of proteins | NCBI ID |
| *Zea mays* | SRR23046702 | 6765236 | *Setaria italica* | 207428 | GCA_000263155.2 |
|  |  |  | *Digitaria exilis* |  | GCA_015342445.1 |
|  |  |  | *Panicum miliaceum* |  | GCA_003046395.2 |
|  |  |  | *Sorghum bicolor* |  | GWHEQHX00000000 |
| *Sorghum bicolor* | SRR34258085 | 6262038 | *Setaria italica* | 218248 | GCA_000263155.2 |
|  | SRR34258119 |  | *Digitaria exilis* |  | GCA_015342445.1 |
|  | SRR34258120 |  | *Panicum miliaceum* |  | GCA_003046395.2 |
|  |  |  | *Zea mays* |  | GCA_902167145.1 |
| *Glycine max* | SRR10305130 | 557147 | *Mucuna pruriens* | 171252 | GCA_003370565.1 |
|  | SRR10309858 |  | *Phaseolus vulgaris* |  | GCA_000499845.2 |
|  | SRR10309859 |  | *Pueraria montana* |  | GWHBEIM00000000 |
|  | SRR10309860 |  | *Vigna radiata* |  | GWHEQVC00000000 |
|  | SRR10309861 |  |  |  |  |
|  | SRR10309862 |  |  |  |  |
|  | SRR10309863 |  |  |  |  |
|  | SRR10309864 |  |  |  |  |
|  | SRR10309865 |  |  |  |  |
|  | SRR10309866 |  |  |  |  |
|  | SRR10309867 |  |  |  |  |
|  | SRR10309868 |  |  |  |  |
|  | SRR10309869 |  |  |  |  |
|  | SRR10309870 |  |  |  |  |
|  | SRR10309871 |  |  |  |  |
|  | SRR10309872 |  |  |  |  |
|  | SRR10309873 |  |  |  |  |
|  | SRR10309874 |  |  |  |  |
|  | SRR10309875 |  |  |  |  |
|  | SRR10309876 |  |  |  |  |
|  | SRR10309877 |  |  |  |  |
|  | SRR10309878 |  |  |  |  |
|  | SRR10309879 |  |  |  |  |
| *Solanum lycopersicum* | SRR24295332 | 1507736 | *Solanum tuberosum* | 181534 | GCA_020169535.1 |
|  | SRR24295336 |  | *Capsicum annuum* |  | GCA_011745845.1 |
|  |  |  | *Nicotiana tabacum* |  | GWHGEXH00000000.1 |
|  |  |  | *Solanum torvum* |  | GWHCAXF00000000 |
| *Solanum tuberosum* | *SRR14298411* | 3976347 | *Solanum lycopersicum* | 180523 | GWHFILF00000000.3 |
|  | *SRR14298438* |  | *Capsicum annuum* |  | GCA_011745845.1 |
|  | *SRR14298440* |  | *Nicotiana tabacum* |  | GWHGEXH00000000.1 |
|  |  |  | *Solanum torvum* |  | GWHCAXF00000000 |


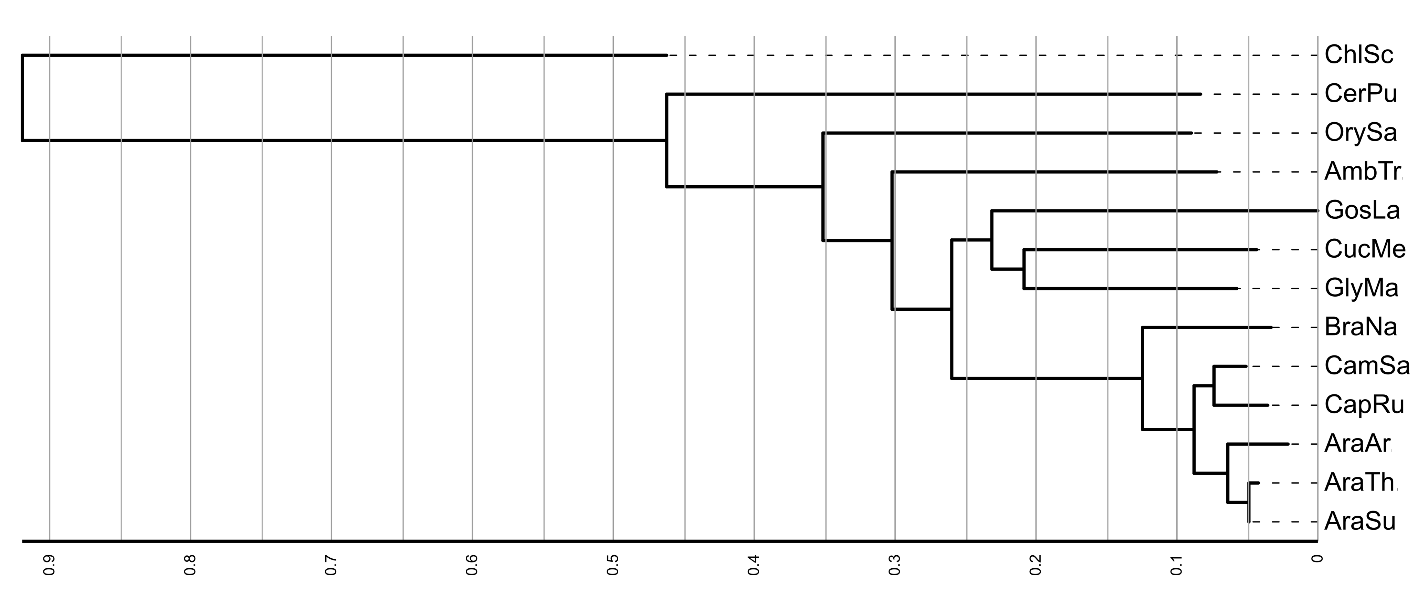


**Figure S1**. Phylogenetic tree of species used as protein sources for the annotation of the *Arabidopsis thaliana* genome: *Arabidopsis thaliana* (AraTh), *Arabidopsis suecica* (AraSu), *Arabidopsis arenosa* (AraAr), *Camelina sativa* (CamSa), *Capsella rubella* (CapRu), *Brassica napus* (BraNa), *Glycine max* (GlyMa), *Cucumis melo* (CucMe), *Gossypium laxum* (GosLa), *Amborella trichopoda* (AmbTr), *Oryza sativa* (OrySa), *Ceratodon purpureus* (CerPu), *Chlamydomonas schloesseri* (ChlSc).
